# A Computational Approach to Hand Pose Recognition in Early Modern Paintings

**DOI:** 10.3390/jimaging9060120

**Published:** 2023-06-15

**Authors:** Valentine Bernasconi, Eva Cetinić, Leonardo Impett

**Affiliations:** 1 Digital Visual Studies, University of Zurich, 8006 Zurich, Switzerland; 2 Digital Society Initiative, University of Zurich, 8001 Zurich, Switzerland; eva.cetinic@uzh.ch; 3 Cambridge Digital Humanities, University of Cambridge, Cambridge CB2 1RX, UK; li222@cam.ac.uk

**Keywords:** annotated dataset, hand pose estimation, hand pose, image classification, digital art history, hand gestures, paintings, early modern times, digital humanities

## Abstract

Hands represent an important aspect of pictorial narration but have rarely been addressed as an object of study in art history and digital humanities. Although hand gestures play a significant role in conveying emotions, narratives, and cultural symbolism in the context of visual art, a comprehensive terminology for the classification of depicted hand poses is still lacking. In this article, we present the process of creating a new annotated dataset of pictorial hand poses. The dataset is based on a collection of European early modern paintings, from which hands are extracted using human pose estimation (HPE) methods. The hand images are then manually annotated based on art historical categorization schemes. From this categorization, we introduce a new classification task and perform a series of experiments using different types of features, including our newly introduced 2D hand keypoint features, as well as existing neural network-based features. This classification task represents a new and complex challenge due to the subtle and contextually dependent differences between depicted hands. The presented computational approach to hand pose recognition in paintings represents an initial attempt to tackle this challenge, which could potentially advance the use of HPE methods on paintings, as well as foster new research on the understanding of hand gestures in art.

## 1. Introduction

Hands represent an important aspect of our mode of communication, and paintings are no exception. Indeed, from medieval representations of symbolic gestures [1] to their more natural depictions in European early modern art [2] (p. 86), hands seem to convey precious information about sociocultural evolution and play a key role in the pictorial narrative system. Although the specific topic of hand gesture analysis has been fairly sparsely addressed in art history and digital humanities, the recent work by the art historian Dimova [3] defines the foundations of a categorization system of painted hand gestures. In total, her system proposes 30 chirograms, each referring to a specific hand pose and its possible significance. This represents a starting point for a new kind of pictorial reading and serves as a basis for the classification problem addressed in this paper. However, there are several important challenges that need to be addressed in order to transform the art historical categorization scheme into a computational workflow. First of all, the art historical categorization of painted hands is very ambiguous and strongly depends on both the context of the depicted scene as well as the context of the creation of the painting. More specifically, in order to correctly classify the hand gesture, one must be familiar with the iconographic theme of the painting as well as the role and the specific position of the figure from which the hand has been extracted. Furthermore, regarding the context of creation of the artwork, in particular, the sociocultural context of the painter itself can be an indication of the gestural convention in place [4]. This indicates the need for deep contextual knowledge in the data annotation process and hinders the possibility of performing crowdsourcing-based data annotation, making it difficult to work with large-scale datasets.

In the last decade, there has been increased interest in exploring computer vision and machine learning methods for analyzing and categorizing digitized collections of paintings. Most of the work in this field is focused on classification [5,6,7,8], pattern recognition and retrieval [9,10,11,12,13], or object detection in paintings [14,15,16,17]. Studying bodily depictions in the context of digital art history has so far been mostly exclusively focused on body pose detection and recognition [18,19,20]. Such works, essentially based on the aforementioned operationalization of theories presented by the famous art historian Aby Warburg [21], ground their methodological approach in human pose estimation (HPE). Based on deep learning models [22,23], trained on large, annotated dataset of real images [24,25], such as OpenPose [22,26], these procedures produce keypoint features that can be used for pose comparison [19,27,28,29]. However, these models achieve a lower accuracy on artworks [20,30], that integrate visual features other than natural images, such as layouts and brush stroke marks. One of the major current challenges is the lack of proper training datasets that would improve the results of applying HPE techniques to paintings. Moreover, while body pose estimation and its iconographic interpretation has been the subject of recent studies [30,31,32,33,34], the task of hand pose classification in paintings has not yet been computationally addressed.

More specifically, computer-vision-based approaches, such as hand gesture recognition (HGR) and classification, have been extensively addressed and defined within the domain of human–computer interaction (HCI) [35,36,37,38]. HCI includes research on the development of sensor-based gesture detection [39], single RGB cameras [40], and depth cameras [41,42,43], as well as the attribution of hand poses with a recent focus on deep learning methods [44,45,46]. From a temporal perspective, hand gestures can be divided into *static* or *dynamic* categories. Static hand gestures are commonly called hand poses, and their understanding relies on their orientation, the positions of the fingers, the angles shaped by their articulations, and their relative positions toward the body [36]. In addition to the temporal perspective, communicative and manipulative gestures are also often differentiated without clear taxonomies defined among the field [35]. Ultimately, gesture recognition systems can be sequenced into different processes, such as identification, tracking, and classification or detection, feature extraction, and classification [35,37].

The field of sign language recognition (SLR) also includes notable research on the development of the live detection and attribution of hand poses [47,48]. Methods mostly involve the analysis of RGB and depth images representing the hand, but also the face and body movements of an individual [47]. Although SLR methods are also applied to static hand poses in the case of alphabets for sign language processing [49,50], the methodology mostly applies to dynamic hand gesture recognition. Furthermore, it seems that iconographic categories of painted hands from early modern times significantly differ from the nomenclature of sign language [3]. Indeed, sign language is a living language, defined by grammatical rules that not only involve the hands but also the face and other body parts [47].

Nevertheless, most of the deep learning models used in this context are trained on large annotated datasets of photographic images and achieve lower accuracy on artworks, which presents new challenges for the field of computer vision.

In this work, we propose the exploitation of methods and body characterization proposed in previous research on digital art history and apply it to the hands. Using HPE methods, we explain the process of the creation of a dataset of painted hands. It includes multiple curatorial steps, which reveals gaps between art historical and computational understanding of digitized artistic material. From the dataset produced, we define different features and propose a classification task based on art historical chirograms. In particular, our main contributions are:

- The first annotated dataset of painted hands (Painted Hand Pose dataset), introducing a new standard for hand pose annotation in the context of digital art history;
- New hand pose features based on 2D keypoint information produced by HPE methods;
- The introduction of a novel classification task and a proposed solution method based on keypoint feature extraction.

Our newly introduced Painted Hand Pose dataset and the proposed keypoints feature represent a first attempt to establish a computational framework for the analysis of hand gestures in paintings. This approach can serve as a basis for future research work or different applications, such as browsing data collections of painting through gestures, as presented in [29]. In the context of this work, we additionally define and explore an automated classification task in order to better understand the potential and limitations of the dataset and features. Because of the small size of the dataset and the complex information encoded within the depiction of various hand poses in paintings, this classification reveals a new possible challenge for the field of computer vision, opening new possibilities for future exploration of this subject.

## 2. Materials and Methods

### 2.1. Dataset

#### 2.1.1. Data Acquisition and Metadata Processing

The data source consists of digital images and their corresponding metadata from the photographic collection of the Bibliotheca Hertziana—Max Planck Institute for Art History in Rome. Because early modern paintings offer a figurative content and an interesting shift between symbolic and more natural hand gestures, we focused on Western European artworks produced between the 15th and the 17th centuries, thus harvesting a total of 5234 images.

**Figure 1:**
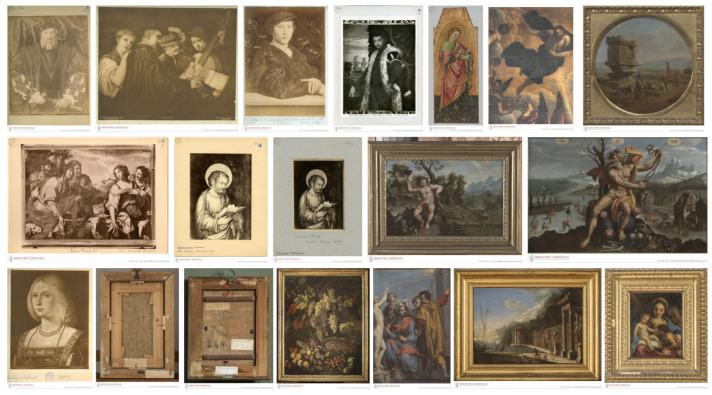
Example images of paintings from the source data collection, the photographic Collection of the Bibliotheca Hertziana, Max Planck Institute for Art History. Reprinted with permission from Bibliotheca Hertziana, Max Planck Institute for Art History in Rome. 2023.

These images are photographs of paintings taken over the past century. Many of them are in black and white, and early acquisitions correspond to documents holding both an image and written information on the bottom, as shown in Figure 1, traditional material used in the practice of art history. Not all paintings represent bodies and hands, as the collection also holds depictions of landscapes, still-lifes, close ups, and portraits, as well as the backs of framed canvases.

The images are associated with their corresponding metadata, which include the title of the paintings, the author, the estimated year of creation, and the identification number. As the digital photographic collection was created by different art historians and the metadata were produced at different time stages, they often include information in different languages as well as date formats following different art historical conventions. Therefore the first step in the preparation of our dataset included processing, cleaning, and standardizing the available metadata.

#### 2.1.2. Hand Extraction

The second step in the creation of the dataset was extracting hands from the collection of paintings. For this purpose, we compared several different approaches based on pretrained convolutional neural network models, such as the DensePose-RCNN [23], handCNN [51] and OpenPose [22,26]. We found that the OpenPose model yielded the best detection results on our dataset of paintings, and therefore, we used this model to create the final dataset of hands. OpenPose represents a real time multiperson system that can detect human body, hand, facial, and foot keypoints on single images. It detects a total of 135 keypoints, with 21 keypoints assigned to each hand, together with an indication referring to the left or right hand. For the purpose of creating our dataset of painted hands, we first applied the OpenPose model on artwork images for multiperson pose extraction. Each keypoint $k_{i}$ was defined by its position coordinates (x,y) and a detection confidence value *c*:

(1) $$k_{i}=\left(x_{i},y_{i},c_{i}\right),\mathrm{where}i\in \left\{0,1,\ldots 20\right\}$$

**Figure 2:**
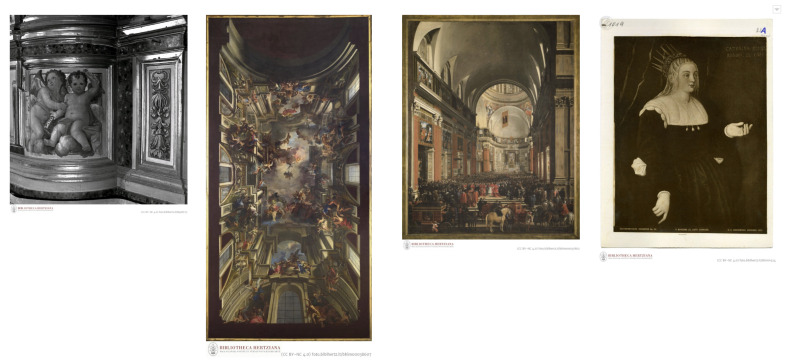
Sample of pictures representing an issue for automated hand detection with OpenPose. Reprinted with permission from Bibliotheca Hertziana, Max Planck Institute for Art History in Rome. 2023.

If no hand was detected for an image, then the coordinates and score value (x,y,c) were set to 0. Out of the 5234 images given as input to the OpenPose model, one or more bodies were estimated on a total of 4256 images. However, the OpenPose model was not able to detect hand keypoints on all of the images that included depictions of bodies. For example, images presenting body depictions within architectural ornamentation, such as walls and ceilings frescos, present a challenge for hand extraction, presumably because of the distortion of the perspective. It is also very difficult to detect the body and hand keypoints in depictions of crowds and in images where the visibility of the body is obscured because the color of clothes or body parts is fused with the color of the background, usually when the image is dark and has low contrast. Figure 2 shows some examples of artwork images on which the OpenPose model failed to detect hands.

**Figure 3:**
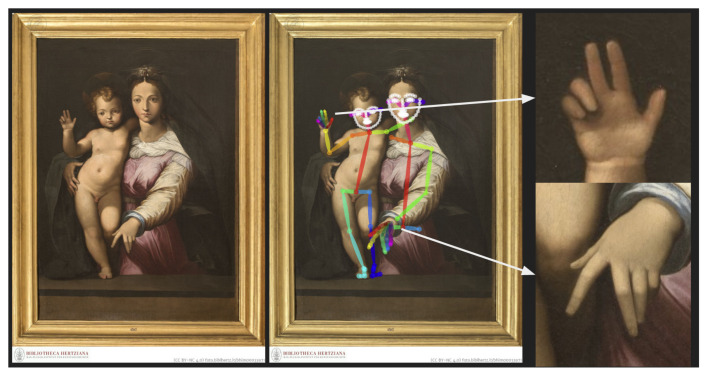
Illustration of the hand extraction process using the pretrained OpenPose model on one artwork image example.

After excluding images without detected hand keypoints, we proceeded to extract the hand images from those artwork images on which the OpenPose model detected hand keypoints for one or more hands. Based on the coordinates used for the keypoint descriptions, a bounding box was generated around the hand in order to crop the image, as illustrated in Figure 3. The bounding box was defined with the minimum and maximum coordinate values, which were first extracted from all *x* and *y* coordinate values of the different hand keypoints. From the minimum and maximum values, the height *H* of the hand was calculated in order to define a proportional margin *m* surrounding the hand. This margin was defined by the height value multiplied by a predefined ratio which was set to a fixed value of 0.3 after visually inspecting the results of different values in several experimental setups.

(2) $$\begin{array}{c} H=y_{max}-y_{min}\\ m=H\times 0.3\\ \left(Box_{min},Box_{max}\right)=\left(\left(x_{min}-m,y_{min}-m\right),\left(x_{max}+m\right),\left(y_{max}+m\right)\right) \end{array}$$

After performing this process on all of the available images, a total of 18,641 cropped images were produced. However, as not all the resulting images actually included hands, manual cleaning of the dataset of cropped images was performed.

During the first visual inspection, cropped images that did not represent a hand were removed from the dataset, which resulted in 15,124 samples. The following step then consisted of checking the quality of the keypoint estimation: the keypoint information was superimposed on each remaining image. This second visual inspection allowed us to remove hands where the keypoints were visibly outside the limits of the depicted hand, as well as left and right hand duplicates, thus resulting in a total of 5993 images of painted hands. Besides the false positives and inaccurate detections, some poses were also more complex to detect and were not recognized by the OpenPose model, which is an issue that has already been documented in related studies [25,30].

#### 2.1.3. Data Categorization and Annotation

The categorization of hands in our dataset was based on an interpretation of the work of the art historian Dimova [3]. In her fundamental work *Le langage des mains dans l’art*, she described a total of 30 hand poses [3] (pp. 320–324), called pictorial chirograms, a notion already introduced by the father of sign language, John Bulwer, in 1644, to illustrate specific hand gestures.

In this work [3], the different hand poses are presented as a lexicon of chirograms, associated with cropped paintings of hands. Although Dimova provided one example image for each hand pose category, each hand pose can indicate several different meanings. Therefore, the categorization of hand pose images is highly ambiguous, as the interpretation of these hands depends on both the context of the depicted scene as well as the overall context of creation of the artwork itself. Furthermore, the context of creation of the artwork, such as, for example, the sociocultural backgrounds of the artists, can be an indication of the gestural convention in place [4]. In order to correctly classify the different hands, expert-level knowledge is therefore required in the data annotation process.

**Figure 4:**
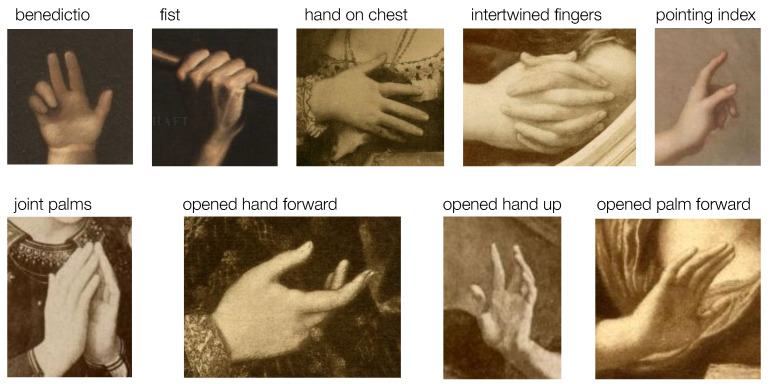
Nine different hand categories based on the chirograms defined by Dimova.

Another challenge for our data labeling process based on Dimova’s categorization is the scarce representativity of specific categories, such as, for example, *thumb up*, *index* and *auricular up*, or *hand under the chin*. Additionally, the variations between different chirograms can be very subtle. In particular, we noticed the case of the pointing finger, which most often indicates a discursive situation, but which can also refer to a higher authority when pointing up [3] (p. 320). We therefore decided to merge all chirograms representing a pointing finger into one category, the *pointing index*. Finally, after excluding all under-represented and ambiguous categories from the chirograms, we restricted our final data annotation task to nine different categories of hand poses, shown in Figure 4.

**Figure 5:**
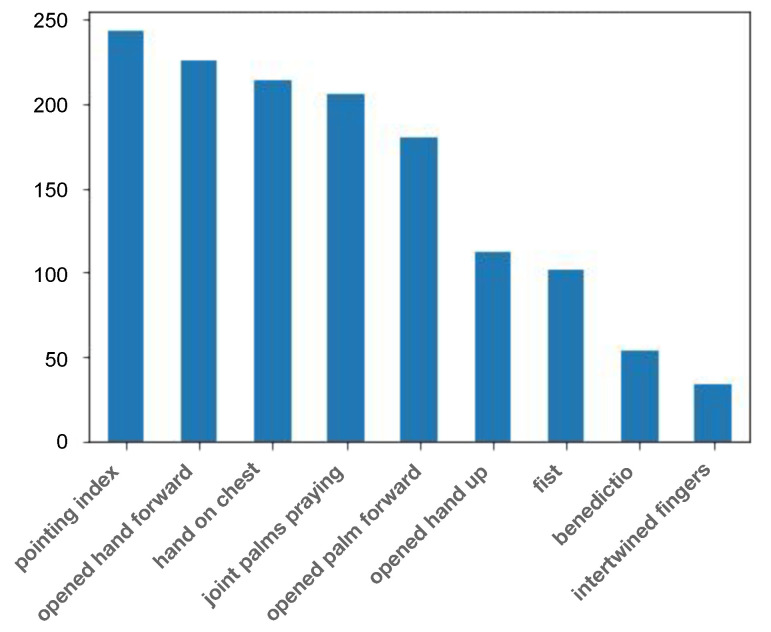
Category-based distribution of images in the Painted Hand Pose dataset.

The images were then manually annotated by the first author of this paper. The final annotated dataset, named the Painted Hand Pose (PHP) dataset, represents 23.3% of the entire dataset of the 5993 initially extracted hand images. The distribution of the number of images into each category can be seen in Figure 5. There is significant variation within the distribution of images per category, e.g., two categories, such as the *benedictio* and the *intertwined fingers*, are associated with less than 100 samples, whereas the *pointing index* has almost 250 samples. However, although this represents a challenge for computational classification, it is a property that is inherent to both the dataset and the categorization task itself, as not all hand poses appear equally frequently in paintings from the early modern times.

The resulting PHP dataset has been made publicly available for future research (https://doi.org/10.5281/zenodo.8069651 (accessed on 24 April 2023)). In order to analyze the potential of this dataset in the context of computational analysis of artwork images, we further explored feature extraction methods and several approaches to the automated classification of the defined hand gestures.

### 2.2. Feature Descriptors

#### 2.2.1. Hand Keypoint Features

**Figure 6:**
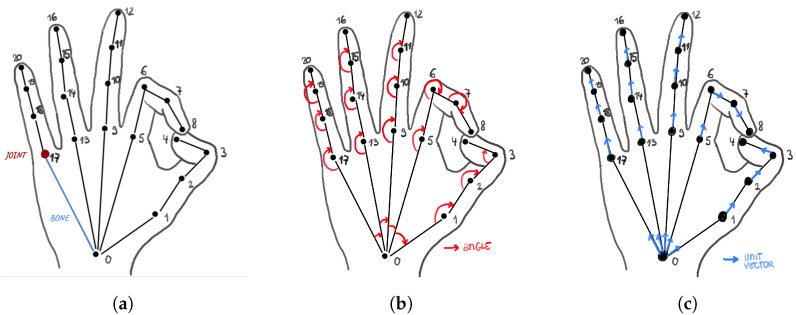
Hand pose representations illustrating the positions of the 21 main OpenPose hand keypoints, which serve as a basis for our hand feature descriptions based on the 19 angles and 20 unit vectors. (**a**) The 21 hand keypoints, (**b**) the 19 hand angles in red, and (**c**) the 20 hand unit vectors in blue.

Based on the hand keypoint coordinates extracted using the OpenPose model, we defined a new set of features for hand pose classification. Figure 6a shows the positions of the 21 hand keypoints together with their defined indices. The OpenPose model outputs a total of 21 keypoints for a single hand, denoted as $k_{i},i=\left[0,\ldots ,20\right]$, from which 1 keypoint corresponds to the base of the palm or the wrist, 5 correspond to the fingertips, and 16 correspond to the articulations of the fingers, also called joints.

Inspired by the work of Impett [28] on body pose comparison, we decided to characterize the hand poses by the angles shaped by the joints and use the values of the angles as feature descriptors of the hand images. Each angle was defined with three keypoints, excluding the fingertips, as shown on Figure 6b. A list of 19 possible angles $\theta_{a,b,c}$ was constructed, where a,b,c correspond to the defined keypoint indices, and the angle $\theta_{a,b,c}$ represents the angle between the central keypoint at position *b* and two neighbouring keypoints *a* and *c*:

(3) $$\begin{array}{c} \theta_{a,b,c}\in \left[\theta_{5,0,1},\theta_{9,0,5},\theta_{13,0,9},\theta_{17,0,13},\theta_{j-1,j,j+1}|j\in Z,1\leq j\leq 19,j\notin \left\{4,8,12,16\right\}\right] \end{array}$$

As we have the coordinates of every keypoint a,b,c, we can calculate the Euclidean distances between them:

(4) $$\begin{array}{c} d_{ab}=\sqrt{{\left(x_{b}-x_{a}\right)}^{2}+{\left(y_{b}-y_{a}\right)}^{2}}\\ d_{ac}=\sqrt{{\left(x_{c}-x_{a}\right)}^{2}+{\left(y_{c}-y_{a}\right)}^{2}}\\ d_{bc}=\sqrt{{\left(x_{c}-x_{b}\right)}^{2}+{\left(y_{c}-y_{b}\right)}^{2}} \end{array}$$

We can then use the distance values to calculate the angle $\theta_{a,b,c}$ by applying the Law of Cosines:

(5) $$d_{ac}^{2}=d_{ab}^{2}+d_{bc}^{2}-2d_{ab}\cdot d_{bc}\cdot cos\left(\theta_{a,b,c}\right)$$

where then the angle $\theta_{a,b,c}$ is given by:

(6) $$\theta_{a,b,c}=arccos\left(\frac{\left(d_{ab}^{2}+d_{bc}^{2}-d_{ac}^{2}\right)}{\left(2d_{ab}\cdot d_{bc}\right)}\right)$$

The calculated angle values indicate the relative positions of the fingers and therefore serve as informative feature descriptors for hand gesture recognition. However, besides the angle values between joints, hand gestures are also characterized by the absolute positions and rotation of the palm and fingers. As previously shown in Figure 4, some hand gesture categories present hands that have similar articulations of joints, but more noticeable differences emerge on the level of hand orientation, such as with the categories *hand on chest* and *praying hands*. The orientation of a finger is also instructive regarding the possible space between fingers when, for example, the fingers are spread apart or stuck together. To represent the information related to the direction and orientation of the hand and fingers, we calculated the unit vectors of segments between a pair of keypoints, denoted as *bones* in Figure 6a.

Each bone is defined by two keypoints, excluding the fingertips, as shown on Figure 6c. A list of 20 possible bones $B_{a,b}$ was constructed, where *a* and *b* correspond to the defined indices of neighboring keypoints:

(7) $$\begin{array}{c} B_{a,b}\in \left[B_{0,5},B_{0,9},B_{0,13},B_{0,17},B_{j,j+1}|j\in Z,0\leq j\leq 19,j\notin \left\{4,8,12,16\right\}\right] \end{array}$$

The unit vector of the bone is represented by a tuple $\left(u_{x_{ab}},u_{y_{ab}}\right)$, where

(8) $$\begin{array}{c} u_{x_{ab}}=\frac{x_{b}-x_{a}}{d_{ab}}\\ u_{y_{ab}}=\frac{y_{b}-y_{a}}{d_{ab}} \end{array}$$

The tuple values of the unit vector for each bone constitutes a total of 40 unit vector features per hand image. These unit vector features are concatenated together with the 19 angle value features, forming the final 59-dimensional feature descriptor, which we refer to as keypoint features (KP features).

#### 2.2.2. Neural-Network-Based Image Features

The hand keypoint features were exclusively based on the hand keypoint information obtained using the OpenPose model. In order to understand the descriptive potential of those features for the task of hand gesture recognition, we compared them to other image-based features. In particular, we extracted feature representations of the hand images from two different pretrained neural network models.

In the first case, we used a ResNet50 model pretrained on the well-known ImageNet dataset [52], which has become a standard in many computer vision-related tasks. The model takes a 224 × 224 resized image as input. We used 2048 × 7 × 7 dimensional outputs of the last sequential layer as the basis for our feature descriptor. The outputs of this layer were flattened into a vector of size 100,352, on which we performed a Principle Component Analysis (PCA) to reduce the dimensionality to 1000. We refer to this 1000-dimensional image representation as the ImageNet ResNet50 feature descriptor.

Additionally, in order to test whether we can leverage the availability of larger sign language datasets for our task, we fine-tuned the same ImageNet pretrained Resnet50 model on a dataset for sign language recognition, called the American Sign Language (ASL) dataset [53]. The dataset consists of images of hands categorized into 29 different classes: 26 different alphabetic letters from American sign language, two extra classes for hand poses indicating *space* and *delete* signs, and a nonhand class, which includes images without hands. The training dataset contains 87,000 images, where each sample is a 200 × 200 pixels image. The fine-tuned model requires a 64 × 64 input shape and the addition of sequential layers with a final softmax activation layer to output a prediction on the 29 possible classes. We fine-tuned the model using the categorical cross entropy loss function and the Adam optimizer for 50 epochs, with a 128 batch size and a learning rate of 0.0001. The model reached 84% accuracy on the American Sign Language (ASL) dataset. After fine-tuning this model, we utilized it to extract features from our Painted Hand Pose dataset. We used the outputs of the last sequential layer, which form a 512-dimensional vector, to refer to this descriptor as the ASL ResNet50FT (fine-tuned) features.

### 2.3. Classification Settings

The classification of the hands was performed on each type of feature and with different machine learning methods. In total, we compared the results of three different classifiers: a Multilayer Perceptron (MLP), a Support Vector Machine (SVM), and a K-Nearest Neighbor (KNN) classifier. For the SVM, a Radial Basis Function (RBF) kernel was used. The parameters of the MLP setup included a 200 batch size, with a maximum of 10,000 iterations, and a 0.001 learning rate. A ReLu activation layer and a limited-memory BFGS (lbfgs) solver for weight optimization were used. In order to train the different classification models and due to the small size of the dataset, a five-fold cross-validation method was used with an 80% and 20% split between the training and testing datasets over each fold. The reported accuracy of the selected models corresponds to the mean of the five scores obtained for each fold.

## 3. Results

### 3.1. Exploratory Analysis

**Figure 7:**
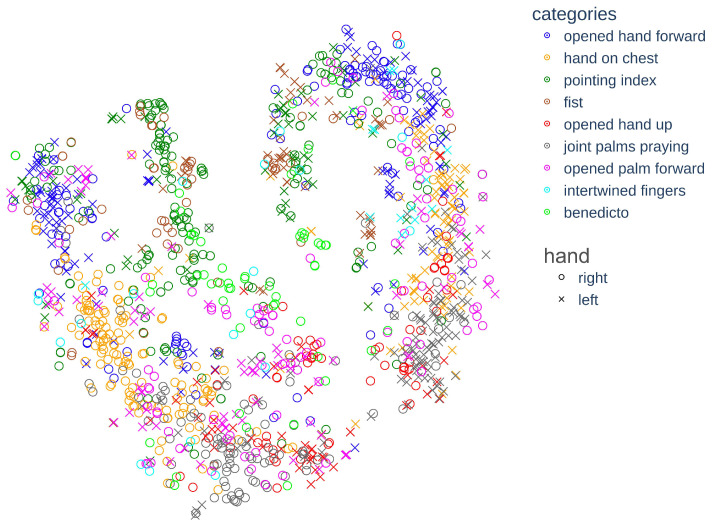
T-SNE projection of the KP features and indication of whether the left or right hand was used.

In order to gain a better understanding of the dataset and the defined class categories, we performed an exploratory analysis which included visualizing the separability of different classes within the dataset. We employed the well-known dimensionality reduction method t-distributed Stochastic Neighbor Embedding (t-SNE) to the KP features extracted from each image in our dataset. The visualization presented in Figure 7 shows each image represented with a 2D point, where the color of the point marks the class category and the shape of the point (circle or cross mark) indicates if it is a right or left hand image. The indication of the right or left hand is based on the OpenPose model keypoint prediction.

The overlapping clusters in the t-SNE visualization indicate that certain classes possess similarities, such as *the pointing index* and the *fist*, as well as the *joint palms praying*, the *hand on chest*, and the *opened hand up*.

**Figure 8:**
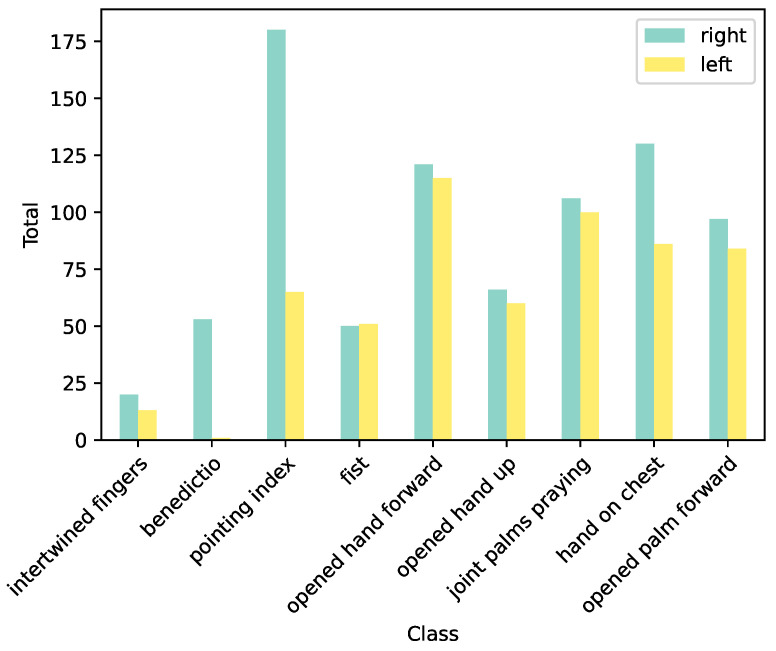
Left and right hand distribution among the categories.

As well as gaining insight about the underlying data structure in regard to the historically defined art categories, our exploratory analysis also focused on general hand categorization aspects, such the relation of the left and right hands. Based on the indication of the left or right hand keypoints, we found that 58% of the hand images in our dataset were labeled as right hands. A more detailed analysis of the distribution of left and right hands within each class category is shown in Figure 8.

As we can see from the results, all gestures, especially the *benedictio*, the *pointing index*, and the *hand on chest*, tended to be mostly performed by the right hand. This tendency for right-handedness in conventional gestures found in early modern paintings correlates with contemporary research on the perception of the right hand as being the dominant hand [54,55]. Furthermore, the reason for the right hand prevalence is also strongly related to the different symbolic connotations of the right and left hands in the early modern period. Indeed, the *benedictio* was only performed by the right hand, and our findings align with historical art observations on the long-standing gestural convention and the liturgical practices from which it derives [56,57]. However, it is important to note that the accuracy of determining whether a hand is left or right using the OpenPose model can vary significantly, especially in the challenging scenarios that emerged in our dataset. Therefore, this exploratory analysis represents only an initial indication and further research is necessary to derive the quantitative relation between left and right hands in early modern paintings with more certainty.

### 3.2. Classification Results

**Figure 9:**
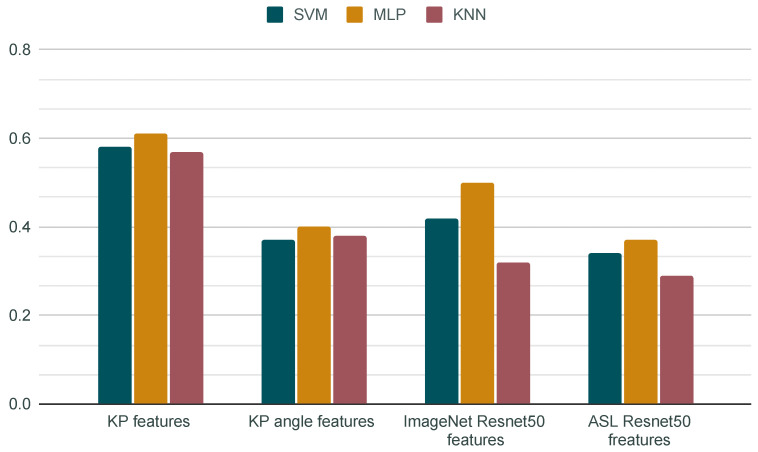
Classification accuracy of different classifiers and features.

The comparison of different feature descriptors based on three different classification methods is shown in Figure 9. The results indicate that our KP features, with a 0.6 accuracy mean obtained using the MLP classifier, outperform the results of the other neural-network-based image features. We also compared the accuracy difference when using all KP features and when using only the KP features related to the angle values. We obtained a significantly lower accuracy when using only the KP angle features, which outlines the importance of the unit vector features for the description of the painted hand gesture as well as confirming the need to encode the direction of the palm and fingers for historical art hand gesture classification.

The 50% accuracy achieved using the MLP classifier on the ImageNet ResNet50 features also indicates a promising result, particularly considering that the model was trained to classify images from very diverse object categories. These features also significantly outperform features extracted from the ResNet50 model fine-tuned on the sign language datasets, with which we achieved an accuracy of only 37% using the MLP classifier. Our initial intuition that we might achieve better results if we fine-tuned a general object recognition model on a sign language recognition task using a specific dataset of hands was therefore proven wrong. As well as the fact that the dataset of photographic sign language hand images is very different to our dataset of painted hands, we also assume that the model trained for the very specific task of sign language classification cannot generalize well to different types of hand poses of early modern paintings because those are, in the end, fundamentally different tasks. This outcome also indicates, once again, the very intricate nature of the computational approach towards painted hand gesture classification and demonstrates the limits of leveraging existing pretrained models and available large datasets of real hand images.

**Figure 10:**
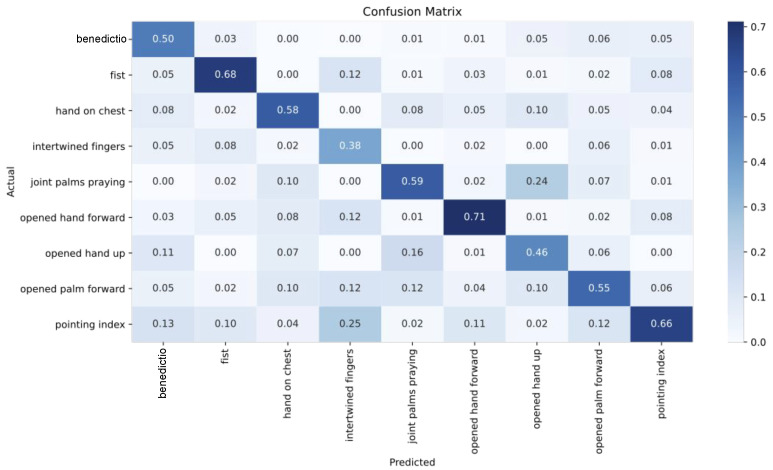
Confusion matrix from the MLP classifier on KP features.

In order to better understand the challenges of painted hand gesture classification, we analyzed the per-class classification performance. The detailed per-class classification results of the MLP are presented with the confusion matrix shown in Figure 10.

**Figure 11:**
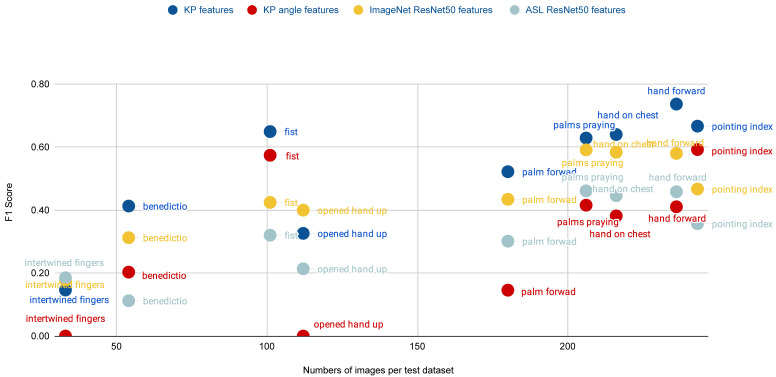
The F1 score value in relation to the number of images in the per-class test set and the different feature types obtained when using the MLP classifier.

The presented results indicate that the best overall results were achieved for the *fist*, the *opened hand forward*, the *joint palms praying*, and the *pointing index* classes. The confusion matrix demonstrates that classes with the largest available number of images in the training set also achieved the highest per-class accuracy scores. This observation was additionally confirmed by the comparison of the F1 score of each set of features with the number of test set images available in each class, as expressed in Figure 11. We found a tendency for better F1 scores based on the number of images in the test set, with the exception of the *fist* class. The high F1 score for the *fist* class reflects the obvious distinctiveness of this hand gesture in comparison to other gestures, mainly as it usually includes a hand with all fingers folded towards the palm.

Additionally, the comparison of the F1 scores of all the different feature types indicates the strength of each feature type on the different classes. For example, the simplified KP features, which included only the angle value information, successfully described the fist and pointing index, but generated low results for all other categories. The ImageNet ResNet50 embeddings also showed good descriptive features for the opened hand up, and the ASL fine-tuned version offered a slightly better representation for intertwined fingers. Overall, it seems that the KP features performed the best for the majority of hand pose classes.

**Figure 12:**
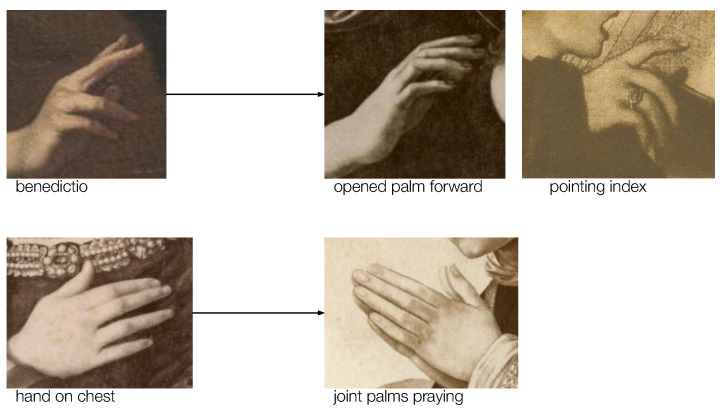
Example images that belong to different classes but depict hands in similar poses.

As well as indicating the well-known problem that occurs when classifying datasets with imbalanced numbers of images per class, the results also demonstrate the impact of a low interclass visual variation. For example, the *benedictio* gesture was often misclassified as *pointing index* and *opened palm forward*, while the *hand on chest* was most commonly misclassified as the *praying gesture* and vice versa. Figure 12 illustrates the complexity of the hand gesture classification tasks, as the differences between hands belonging to different categories are often very subtle. Our in-depth review of the misclassified samples also showed that an issue often emerges when hands present an interplay with an object or with another hand.

### 3.3. Application for Gesture-Based Image Search

**Figure 13:**
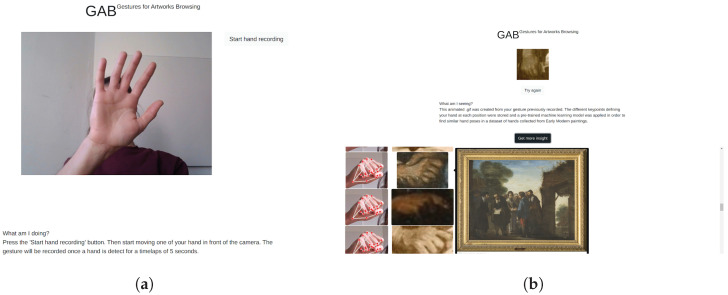
Gestures for Artworks Browsing application. (**a**) Input: real-time recording of the hand, (**b**) Output: similarly painted hand images and their source artworks. Screenshot of the Gestures for Artworks Browsing application.

Finally, it is important to indicate that our dataset and the defined hand keypoint features can be utilized beyond the scope of the presented classification task. More specifically, the defined hand keypoint features serve as a basis for the gesture-based image search engine Gestures for Artworks Browsing (GAB), which is presented and described in more detail in [29]. This application represents an unique framework in the context of digital art history, as it enables the exploration of collections of digitized artworks without using the conventional methods of word- or image-based searches. Figure 13 illustrates the main functionalities of GAB. The code of the GAB implementation is publicly available (https://github.com/VBernasconi/GAB_project (accessed on 22 March 2022)).

**Figure 14:**
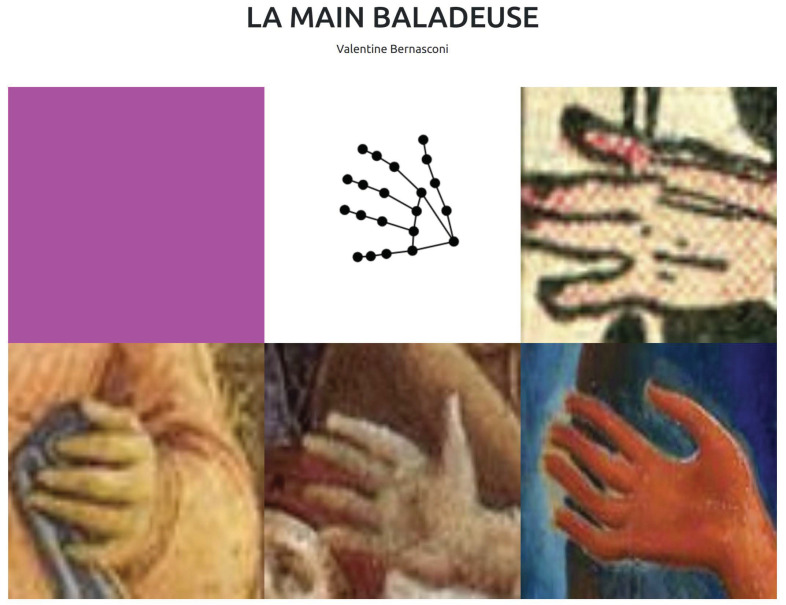
Interactive interface from the art installation *La main baladeuse*, with the hand of the user represented as a skeleton in the center.

As shown in Figure 13, the hand pose of the user is used to retrieve similar hands in the database of paintings and access their corresponding original images. The implementation of the browsing tool uses the KP features and the k-NN algorithm to find the most similar painted hands in relation to the image taken from the user’s hand. This application demonstrates the efficiency of the KP features as a pose descriptor in a context-independent environment. Furthermore, KP features have also been used for real-time interactions in the digital work of art called *La main baladeuse* [58] presented in the digital exhibition of the museum Le Jeu de Paume from May to December 2022. The concept of the work was based on the simultaneous interplay of similarly drawn and painted hands from different time periods and the continuous gestural hand expression of the user facing the camera, as illustrated in Figure 14.

An application such as the Gesture-Based Browsing Interface can be used in a research context to easily access specific painted hands, but it can also be used as a novel and unconventional way of exploring art collections in museum spaces, offering a new experience of the artworks. Combined with a classification model trained for the recognition of pictorial early modern hand gestures, the tool could potentially become a didactic instrument, employed to better understand the diversity of depicted hands and their various connotations in early modern paintings.

## 4. Discussion

To summarize, in this paper, we introduced a new dataset of hand images extracted from paintings from the European early modern period, named the Painted Hand Pose dataset. We presented, in detail, the various challenging steps in the dataset creation process, which highlight the complexity of working with digitized collections of artworks. The dataset creation process included significant manual cleaning effort as well as laborious categorization and annotation work that required historical art expertise.

As well as introducing a novel dataset, we proposed a new feature extraction method based on the use of deep learning methods for human pose estimation. More specifically, we focused on the hand keypoint coordinates obtained using the OpenPose model. These keypoints were used as the basis for our hand keypoint feature descriptor which integrates information about the absolute and relative positions of the palm and fingers by combining the angle values between the different joints in the hand and unites vector values of the different bones of the hand.

Those features serve as the basis for additional computational exploration. For this purpose, in the context of our paper, we introduced a new challenging and interdisciplinary classification task. The work of the art historian Dimova on the categorization of hand gestures in paintings served as a basis for defining our classification task and identifying the different hand pose classes. In this paper, we propose a classification setting in order to better understand the complexity of hand pose classification in the context of artworks as well as to compare our keypoint features with other neural-network-based image features. The features created from the HPE 2D keypoint information showed promising results for this classification task and demonstrated the importance of the angles shaped by the articulation of the fingers and the orientation of the hand as descriptors.

The presented classification results primarily serve to illustrate the complexity of adopting a computational approach towards hand pose recognition in early modern paintings. There are various possibilities for improving the classification results that might be explored in the future, such as exploring different feature ensemble techniques or including features extracted from deep neural network models fine-tuned on different tasks. However, the goal of this work was not to find the best possible classification method but to introduce a novel and challenging interdisciplinary task. The subtle differences between hand poses belonging to different historical art gestural categories outline the importance of integrating additional contextual information. Therefore, another possible future extension of this work includes combining hand pose image features with other global image scene features or existing metadata information (e.g., the title of the painting) in a multimodal setting.

Possible future improvements of the classification results also include the augmentation of the dataset, although this would require manual human effort and expert-level knowledge on hand gestures in the early modern times. There is, however, the possibility of augmenting the existing dataset with synthetically generated images by using generative models such as variational autoencoders, generative adversarial networks, or diffusion models. As well as the issue of homogenization, where not enough diverse data are produced, it seems that hands also represent a great challenge for contemporary text-to-image generative diffusion models, as reported in various media [59,60]. These examples also highlight our own conclusion that hand gestures represent a particularly difficult subject for computational image understanding, even for the most advanced contemporary deep neural network models.

Finally, our newly introduced classification task represents an interesting challenge in the context of the emerging discipline of digital art history. It also contributes to existing challenges in the field of computer vision and hand gesture recognition. Our research represents the first step in the direction of computational understanding of hand poses in paintings. It will, therefore, hopefully foster not only new methods in the context of computer vision, but also new research work on art history that will led to a better understanding of the language of hands in art, the comprehension of various gestural patterns, and their evolution in early modern times.

## Data Availability

The Painted Hand Pose dataset is publicly available at https://doi.org/10.5281/zenodo.8069651 (accessed on 24 April 2023).
